# Peripheral blood gene expression signatures which reflect smoking and aspirin exposure are associated with cardiovascular events

**DOI:** 10.1186/s12920-017-0318-6

**Published:** 2018-01-12

**Authors:** James A. Wingrove, Karen Fitch, Brian Rhees, Steven Rosenberg, Deepak Voora

**Affiliations:** 1 CardioDx, Inc, 600 Saginaw Dr., Redwood City, CA 94063 USA; 2 Center for Applied Genomics & Precision Medicine, Department of Medicine, Duke University, 101 Science Drive, 2187 CIEMAS, Durham, NC 27708 UK

**Keywords:** Gene expression, Smoking, Aspirin exposure, Cardiovascular events

## Abstract

**Background:**

Cardiovascular disease and its sequelae are major causes of global mortality, and better methods are needed to identify patients at risk for future cardiovascular events. Gene expression analysis can inform on the molecular underpinnings of risk factors for cardiovascular events. Smoking and aspirin have known opposing effects on platelet reactivity and MACE, however their effects on each other and on MACE are not well described.

**Methods:**

We measured peripheral blood gene expression levels of ITGA2B, which is upregulated by aspirin and correlates with platelet reactivity on aspirin, and a 5 gene validated smoking gene expression score (sGES) where higher expression correlates with smoking status, in participants from the previously reported PREDICT trial (NCT 00500617). The primary outcome was a composite of death, myocardial infarction, and stroke/TIA (MACE). We tested whether selected genes were associated with MACE risk using logistic regression.

**Results:**

Gene expression levels were determined in 1581 subjects (50.5% female, mean age 60.66 +/−11.46, 18% self-reported smokers); 3.5% of subjects experienced MACE over 12 months follow-up. Elevated sGES and ITGA2B expression were each associated with MACE (odds ratios [OR] =1.16 [95% CI 1.10–1.31] and 1.42 [95% CI 1.00–1.97], respectively; *p* < 0.05). ITGA2B expression was inversely associated with self-reported smoking status and the sGES (*p* < 0.001). A logistic regression model combining sGES and ITGA2B showed better performance (AIC = 474.9) in classifying MACE subjects than either alone (AIC = 479.1, 478.2 respectively).

**Conclusion:**

Gene expression levels associated with smoking and aspirin are independently predictive of an increased risk of cardiovascular events.

**Electronic supplementary material:**

The online version of this article (10.1186/s12920-017-0318-6) contains supplementary material, which is available to authorized users.

## Background

Cardiovascular and cerebrovascular disease (CVD) and their complications continue to be a leading cause of morbidity, mortality, and cost in both the United States and world-wide [1]. Despite new therapies and advanced diagnostic modalities, much remains to be done to mitigate the consequences of this disease. CVD is a complex, multifactorial disease; its development and progression is driven by the interplay of genetics, environment, and diet. Furthermore, the transition from stable, atherosclerotic disease to a major adverse cardiovascular event (MACE, e.g. unstable angina, myocardial infarction, or stroke) is poorly understood though it is likely to be driven, in part, by platelet mediated thrombosis of the coronary or cerebral vessels [2–4]. The biologic pathways and genes that underlie susceptibility to cardiovascular events are only beginning to be described. Genome wide association studies are unraveling the genetic architecture of such events [5, 6] but are limited in the consideration of environmental effects.

An alternative approach to understanding MACE biology has been to study biologic responses to known environmental stimuli that are associated with CVD. Genomic technologies that interrogate genes, transcripts, proteins, and metabolites at the genome-wide level hold the promise of identifying novel disease genes and pathways as they are dynamic and thus integrate the effects of the genome and environmental triggers [7]. Such an approach may help to elucidate the biologic pathways perturbed by environmental exposures known to affect CVD, and thus help reduce the complexity of diseases such as CVD. A variety of these types of studies have been performed to date using dietary [8], pharmacologic [9], psychological [10], and environmental stressors [11] and have identified genes, pathways, and potential biomarkers for CVD. Measuring the levels of genomic markers that reflect specific environmental exposures within an individual may provide a molecular “snapshot” of their CVD risk.

Cigarette smoking has long been identified as a major risk factor for the development of cardiovascular disease and MI [12]. Smoking promotes a pro-atherogenic state through vasomotor dysfunction, increased inflammation, and the generation of a higher-risk lipid profile through increased amounts of oxidized LDL [12]. In addition, smoking produces a pro-thrombotic state driven by increased platelet dysfunction [13, 14] and other hypercoagulable effects [15]. Heightened platelet reactivity, independent of smoking, is also a risk marker for platelet-mediated thrombotic events such as myocardial infarction (MI) and stroke [3, 4]. Aspirin is a potent inhibitor of cyclooxygenase–1 (COX1), leading to decreased production of thromboxane B2 which in turn inhibits platelet function [16]. Although a major pathway for aspirin’s effect on platelet aggregation, there are likely effects of aspirin beyond COX-1 on platelet function, though there is limited understanding of these additional effects [17]. Smoking is also known to influence the inhibitory effects of aspirin on platelet function, with smokers exhibiting higher residual platelet reactivity compared to nonsmokers on aspirin [18]. While aspirin use and smoking status are easy to obtain from patients, capturing their biologic effects on thrombotic risk is challenging in the clinical setting due the technical challenges associated with platelet function testing [19].

In prior work we discovered and validated peripheral blood gene expression signatures for cigarette smoking [20] and antiplatelet therapy with aspirin [9], two specific environmental exposures known to affect platelet aggregation, CVD, and risk for MACE. Briefly, the aspirin response signature [9] is a set of 62 predominantly platelet derived genes, identified in peripheral blood that is correlated with aspirin’s inhibitory effects on platelet function and MACE events, and is likely regulated by a transcriptional mechanism in megakaryocytes [21]. ITGA2B is a prominent member of the aspirin response signature and can be used as a surrogate for the remaining genes in this signature [9]. We have also previously described the development and validation of a 5 gene peripheral blood smoking gene expression score (sGES) that can classify smoking status in patients at risk for cardiovascular disease [20]. Given the potential molecular interactions between smoking, aspirin, and platelet reactivity, we investigated the use of these gene expression signatures as predictors of MACE risk in a population of patients referred for elective, invasive coronary angiography [22].

## Methods

### Patient population and definitions

The smoking GES and ITAG2B expression levels were measured in baseline samples from 1581 participants of PREDICT, a previously described prospective US multi-center observational study of patients referred for coronary angiography (http://www.clinicaltrials.gov, NCT00500617, prospectively registered at clinicaltrials.gov on July 11, 2007) in which angiograms were performed based on local, institutional protocols [22]. The primary endpoint in the present study was MACE, which was an adjudicated outcome in PREDICT and defined as all-cause death, MI, stroke, or transient ischemic attack (TIA). As 15 subjects with MACE lacked exact time-to-event data (although time of visit, either 6 months or 1 year, was retained), events occurring within a one year window from time of coronary angiography were modeled as binary responses and linear predictors using logistic regression. In 69% of the subjects in this study, the presence of obstructive coronary artery disease (CAD) was determined by quantitative coronary angiography (QCA) in which obstructive disease was defined as 1 or more atherosclerotic plaques in a major coronary artery (lumen diameter ≥ 1.5 mm) causing 50% or greater luminal diameter stenosis. For the remaining subjects the amount of stenosis was determined by the clinical site read, and obstructive disease was defined as a 70% or greater occlusion. The institutional review boards at all centers approved the study, and all patients gave written informed consent. Self-reported aspirin and smoking status (never smoked *n* = 696, former smokers = 599, current smokers = 286) were collected in all participants.

### Blood collection, RNA purification, and RT-qPCR

Whole blood samples were collected in PAXgene tubes prior to coronary angiography. PAXgene tubes were processed according to the manufacturer’s instructions, and then frozen at −20 °C. RNA was purified from 400ul PAXgene solution using the magnetic bead-based Agencourt RNAdvance system. PCR primers used for RT-qPCR have been previously described for ITGA2B [9]; primers for the genes in the smoking GES can be found in the Additional file 1: Table S2 RT-qPCR was performed using the Roche Light Cycler 480 system.

### Statistical methods

All statistical methods were performed using either the R software package, v. 2.09 [23] or Minitab, v. 15.1.3. Univariate analysis was performed on key clinical variables, as shown in Table 1. Receiver-operating characteristic (ROC/AUC) analysis and logistic regression were performed to assess the predictive performance of the smoking GES in this cohort. Multivariable logistic regression was utilized to assess associations between age, sex, levels of the GES and ITGA2B, and MACE, with subsequent addition of oCAD status, self-reported smoking status, aspirin use, and platelet count as predictors. Aikman Information Criteria (AIC) was calculated to assess model strength. ITGA2B expression levels were normalized to the mean expression levels of TFCP2 and HNRPF, as previously described [22]. For ease of presentation and interpretation, the RT-qPCR data associated with ITGA2B expression levels were multiplied by −1 so that higher value equated to higher expression levels. As previously published, the smoking GES includes the gene expression levels of 5 genes (CLDND1, LRRN3, MUC1, GOPC, LEF1) used in the following model: (pr(Smoker)/(1-Pr(Smoker)) = 15.78306 + 0.3876 * SEX – 3.3368 * CLDND1–3.4034*LRRN3–1.4847 *MUC1 + 5.9209 * GOPC +2.27166 * LEF1 where SEX =1 if male, 0 if female [20].

**Table 1:** Clinical Demographics

Characteristic	Levels	Overall	No MACE	MACE	*P*-value
N		1581	1526	55	
Female		50.5% (798)	50.9% (777)	38.2% (21)	0.0741
Age (yrs)		60.66 ± 11.46	60.51 ± 11.40	64.92 ± 12.49	0.0125
Race/Eth White non-hisp		88.9% (1406)	89.1% (1359)	85.5% (47)	0.3817
Chest Pain					0.2655
	Typical	42.1% (664)	41.8% (637)	49.1% (27)	
	Atypical	26.2% (414)	26.6% (405)	16.4% (9)	
	Non-Cardiac	2.0% (32)	2.1% (32)	0.0% (0)	
	None	29.7% (469)	29.5% (450)	34.5% (19)	
Hypertension		69.2% (1084)	68.8% (1041)	78.2% (43)	0.1802
Dyslipidemia		69.4% (1053)	68.9% (1011)	82.4% (42)	0.0442
Aspirin		60.7% (959)	60.8% (928)	56.4% (31)	
BMI		30.64 ± 6.71	30.67 ± 6.73	29.82 ± 6.15	0.3189
Systolic BP (mmHg)		136.35 ± 19.34	136.19 ± 19.30	140.74 ± 20.12	0.1074
Diastolic BP (mmHg)		78.71 ± 11.46	78.76 ± 11.40	77.37 ± 13.01	0.4422
Diabetic		10.8% (170)	9.8% (150)	36.4% (20)	< 0.0001
Current smoker		18.5% (286)	18.2% (272)	25.9% (14)	0.1552
Max Stenosis		38.61 ± 39.39	38.07 ± 39.38	53.64 ± 36.84	0.0032

## Results

The multi-center PREDICT trial enrolled patients who had no known CAD and had been referred for coronary angiography [22]. The baseline characteristics for the subset used in this study of 1581 subjects were typical of a population referred for invasive coronary angiography (50.5% female, 11.1% non-White, mean age 60.66 +/− 11.46, Table 1). Of these, 286 (18%) of the patients were self-reported smokers, and the majority reported using aspirin on a daily basis (60.7%, *n* = 959). The incidence of MACE was 3.5% (*n* = 55) during the 12 month follow-up window (Death = 13, MI = 17, Stroke/TIA =25). Age, dyslipidemia, diabetic status, and maximum stenosis on angiography were significantly associated with MACE when assessed independently (Table 1). Neither self-reported smoking nor aspirin use showed a significant association with MACE in univariate analyses (Table 1).

The smoking GES was significantly associated with self-reported smoking status in the full cohort of 1581 subjects (OR = 2.29, 95% CI 2.09–2.52, *p* < 0.001) and in a naïve subset of the patients which had not been used in the previous development or validation of this sGES [20] (*n* = 1200;, OR = 2.19, 95% CI = 1.97–2.42, p < 0.001). In addition, the sGES showed strong classification of smoking status in both cohorts as measured by AUC (Full cohort: AUC = 0.877, 95% CI 0.860 to 0.893; naïve cohort: AUC = 0.864,95% CI 0.860 to 0.893, respectively; Fig. 2) validating our previous work demonstrating that the smoking GES can serve as an in vivo surrogate for the biologic effects of smoking. In a sex and age adjusted model, the smoking GES was significantly associated with MACE (OR = 1.16, 95% CI 1.10 to 1.31, *p* < 0.05), adjusting for oCAD did not affect this association however adjusting for current smoking status marginally attenuated it (Table 2). The association between sGES and MACE differed by smoking status. When stratified by current smoking status, the sGES was significantly associated with MACE in current smokers (*n* = 286, OR = 1.46, 95% CI 1.1 to 1.97, *p* < 0.05) in an age, sex adjusted model but not in non-smokers (Table 2).

**Table 2:** Smoking GES MACE Interactions

Variable	OR (CI)	OR (CI) with oCAD	OR (CI) with Current Smoker	OR (CI) Non- Smokers (*n* = 1259)	OR (CI) Smokers (*n* = 286)
SEX	1.61 (0.92–2.86)	1.58 (0.87–2.9)	1.71 (0.97–3.08)	1.96 (1.01–3.92)	1.38 (0.44–4.7)
AGE	1.05*** (1.02–1.07)	1.04 (1.02–1.07)*	1.05*** (1.02–1.08)	1.05** (1.02–1.08)	1.07* (1.01–1.14)
sGES	1.16* (1.10–1.31)	1.15 (1.014–1.31)*	1.13 (0.97–1.31)	1.01 (0.84–1.21)	1.46* (1.1–1.97)
oCAD		1.4 (0.77–2.5)			
CURRENT SMOKER			1.47 (0.66–3.14)		

*OR* odds ratio, *CI* 95% confidence interval; *** = *p* < 0.001; ** = *p* < 0.01; * = *p* < 0.05

**Fig. 1:**
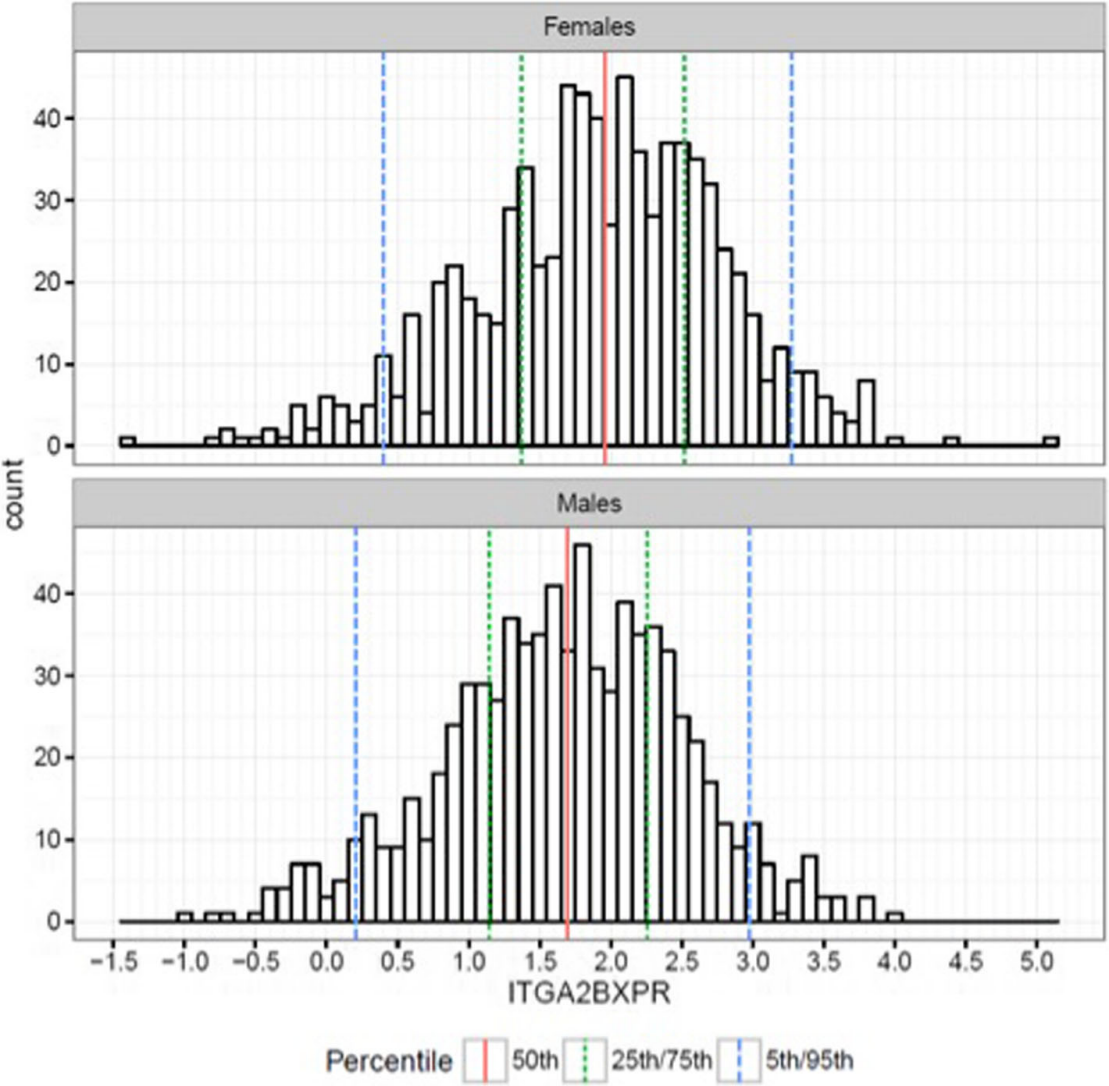
Distribution of ITGA2B gene expression in Females and Males. Median is represented by solid red lines, 25/75th percentile by small dashed green lines and 5th and 95th percentile by large dashed blued lines respectively. Expression values have been transformed from Cp values, so that higher values on the x-axis represent higher expression levels

ITGA2B gene expression was significantly higher in females than males (OR = 1.40, *p* < 0.001, Fig. 1). In a sex and age adjusted model, ITGA2B gene expression displayed a significant association with MACE and remained significantly associated with MACE after adjusting for aspirin use (OR = 1.42, 95% CI = 1.0 to 1.97, *p* < 0.05, Table 3). As ITGA2B is strongly associated with platelet count [9] we further adjusted for this variable, however this did not alter the results (data not shown); adjusting for the presence of oCAD however did diminish the association (Table 3). Because the aspirin response signature is associated with aspirin’s effects on platelet function, we stratified our analysis by aspirin use and found ITGA2B expression was not significantly associated with MACE in non-aspirin users (*n* = 622) and trended to an association when restricted to current aspirin users (*n* = 959) (Table 3).

**Table 3:** ITGA2B MACE Interactions –

Variable	OR (CI)	OR (CI) with oCAD	OR (CI) with Aspirin	OR (CI) No reported Aspirin use (*n* = 622)	OR (CI) Aspirin use (*n* = 959)
SEX	1.95* (1.12–3.49)	1.89* (1.03–3.46)	1.98* (1.13–3.54)	3.24* (1.36–8.57)	1.4 (0.67–2.98)
AGE	1.04** (1.01–1.06)	1.03* (1.00–1.06)	1.04** (1.01–1.06)	1.02 (0.99–1.06)	1.05** (1.02–1.09)
ITGA2B	1.42* (1.00–1.97)	1.36 (0.96–1.92)	1.41* (1.01–1.96	1.23 (0.75–2.04)	1.54 (0.98–2.44)
oCAD		1.44 (0.79–2.60)			
ASPIRIN			0.79 (0.46–1.38)		

*OR* odd ratio, *CI* 95% confidence interval; ** = *p* < 0.01; * = *p* < 0.05

Due to the known interactions between smoking and aspirin responsiveness, we tested the hypothesis that ITGA2B expression and the smoking GES would additively contribute to MACE prognosis. We found an inverse correlation between ITGA2B expression and smoking status (OR = 0.67 [CI = 0.57–0.78]; *p* < 0.001) and the smoking GES (*r* = −0.126; *p* < 0.001, Fig. 3); these relationships were independent of age, sex, presence of oCAD and platelet count. When included together in an age and sex adjusted model, both the smoking GES and ITGA2B gene expression remained independently associated with MACE (Table 4). A single model combining the sGES and ITGA2B was better at explaining the variability in the data (Akaike information criterion, AIC = 474.9) than either alone (AIC = 479.1, 478.2 respectively). To test whether other clinical co-variates might account for the observed association, in a model including the Framingham Risk Score (FRS) both the sGES and ITGA2B gene expression retained significant association with MACE (Table 4). To explore a potential relationship between the smoking GES and ITGA2B gene expression, a model was constructed to assess potential interactions between the GES and ITGA2B; however no significant interaction was observed (Table 4).

**Table 4:** Smoking GES, ITGA2B, FRS and MACE Interactions

Variable	OR (CI)	OR (CI) with FRS	OR (CI) with + sGES:ITGA2B
SEX	1.74 (0.99–3.13)	1.35 (0.69–2.69)	1.74 (0.99–3.12)
AGE	1.04*** (1.02–1.07)	1.03 (1.0–1.06)	1.04*** (1.02–1.07)
sGES	1.17* (1.02–1.32)	1.14* (1.0–1.30)	1.17 (0.85–1.56)
ITGA2B	1.43 (1.03–2)	1.45* (1.04–2.04)	1.45 (0.3–7.60)
FRS		1.04 (0.99–1.09)	
sGES:ITGA2B			1 (0.87–1.16)

*OR* odd ratio, *CI* 95% confidence interval; ** = *p* < 0.01; * = *p* < 0.05

## Discussion

The transition from stable, asymptomatic atherosclerotic cardiovascular disease to unstable disease is poorly understood and is in part a platelet-mediated thrombotic event of a coronary or cerebral artery leading to myocardial infarction or stroke, respectively. Although the degree of atherosclerotic stenosis in a given vessel is well- known to correlate with future risk of MACE, plaque composition and so called “vulnerable plaque” also plays a central role [24]. Similarly, the concept of “vulnerable blood”, a condition in which elevated prothrombotic potential exists in an individual, has been posited for many years. A number of factors contribute to this increased potential, including age, sex, diabetes, race, renal function, and smoking. Conversely, the use of therapeutics such as aspirin and platelet P2Y12 inhibitors has become standard of care in treating platelet hyperactivity associated with acute coronary syndromes and subsequent MACE. However, the biologic pathways affected by aspirin and smoking are not well described nor is the extent to which they are related to MACE. Using a large, multi-center clinical trial population of patients referred for elective coronary angiography (PREDICT) with well-characterized outcomes, we tested the hypothesis that pro- and anti-thrombotic gene expression signatures are associated with MACE. We found that both smoking and aspirin response signatures were associated with MACE, independent of each other and with traditional risk factors.

**Fig. 2:**
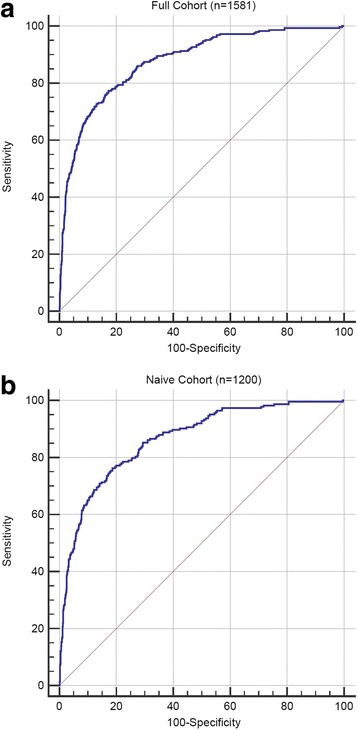
AUC of GES for prediction of self-reported smoking status in full cohort (**a**; AUC = 0.877, 95% CI 0.860 to 0.893) and novel cohort (**b**;: AUC = 0.864,95% CI 0.860 to 0.893)

Cigarette smoking is a well-recognized risk factor for cardiovascular events and has been associated with heightened platelet aggregation and venous and arterial thrombosis for over fifty years [14]. We and others have previously demonstrated a strong association between peripheral blood gene expression and cigarette smoking. Utilizing gene expression profile data from over 1000 subjects, we constructed a 5 gene smoking expression score (sGES) predictive of self-reported smoking status, which we previously validated in 180 independent subjects with an AUC of 0.82 (sensitivity = 0.63; specificity = 0.94 utilizing a 50% probability of smoking as a cutoff) [20]. In this study we further validated the sGES in an independent group of PREDICT trial patients not studied previously and demonstrated a similar ability to identify smokers as in the derivation studies (Fig. 2). PREDICT patients were relatively low risk with only 3.5% developing MACE during the 12 month follow up period and 37% with obstructive CAD by coronary angiography at baseline. Despite these low risk features, we observed that the smoking GES was associated with MACE, independent of the presence of oCAD. In a stratified analysis, the association between the smoking GES and MACE appeared restricted to current smokers, suggesting the sGES is a marker of the biologic response or dose response to smoking with some patients having a heightened cardiovascular hazard of cigarette smoking than others. The association of the sGES with MACE was primarily driven by smoking status; adjusting for Framingham Risk Score which incorporates other clinical risk factors for MACE did not affect the association (Table 4).

Platelet aggregation studies conducted ex vivo have long been used to reflect the capacity for platelets to aggregate in vivo and are correlative of cardiovascular events and although informative, have limited clinical use as they need to be performed on fresh samples [3, 4, 19]. We have previously shown that a peripheral blood gene expression aspirin response signature (ARS), is associated with aspirin’s effects on platelets and is associated with death/MI [9]. Of the genes identified in the ARS, we chose ITGA2B for the present study as it showed the strongest association with aspirin response and was independently associated with MACE [9]. ITGA2B encodes a subunit of the alpha-IIb/beta-3 integrin cell adhesion receptor for fibrinogen, plays a critical role in mediating platelet aggregation, and is the target of several platelet inhibitor medications. In the current study we validated our previous observation, demonstrating an association between peripheral blood ITGA2B expression and MACE. Adjusting for platelet count, aspirin use, smoking status, or FRS did not alter this association. Although adjusting for oCAD widened the precision of the association between ITGA2B and MACE (i.e. wider 95% confidence interval) the point estimate was similar (1.41 vs. 1.36, Table 3) suggesting an independent association with larger sample size. Stratifying by aspirin use appeared to show a stronger effect in aspirin users vs. not (Table 3), which is consistent with the hypothesis that aspirin response signature genes report on the effects of aspirin on platelet inhibition and with our prior data demonstrating an association between aspirin response genes and platelet function only in the presence of aspirin [9]. This apparent diminished effect in non-aspirin users however may also be partially driven by decreased power to detect interactions due to sample size, as adjusting for aspirin usage did not affect the association between ITGA2B and MACE.

**Fig. 3:**
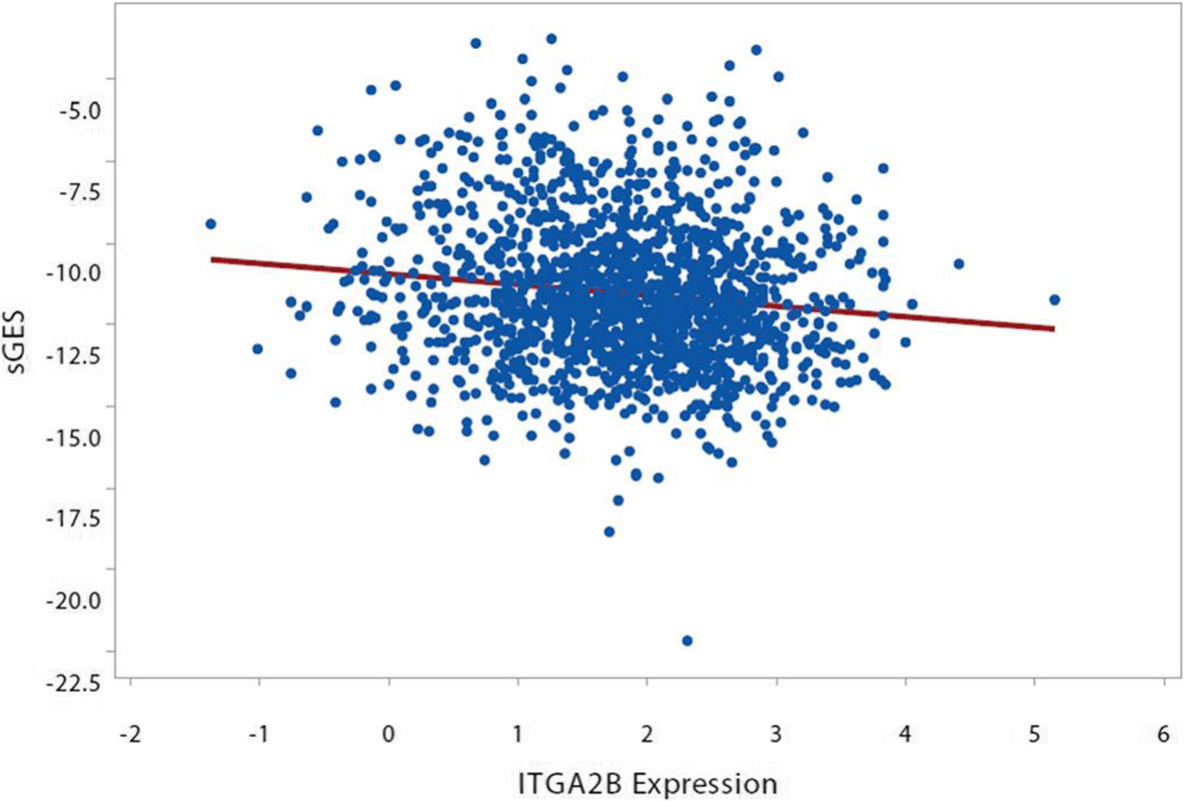
Scatterplot of expression levels of ITGA2B (x axis) versus the sGES (y axis) across the 1581 subjects in the study. A weak but significant negative correlation (r = −0.126; *p* < 0.001) existed between ITGA2B levels and the sGES (red line in graph)

**Fig. 4:**
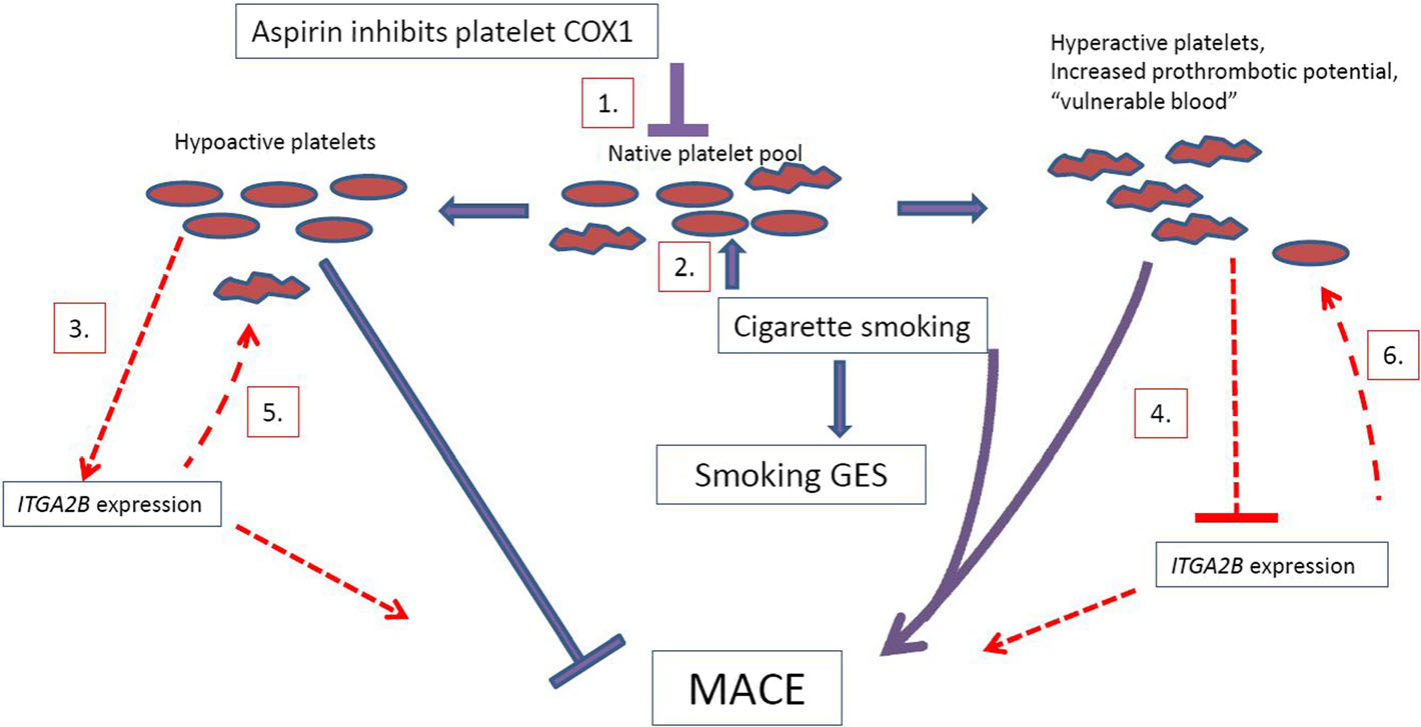
Primary and secondary effects of aspirin, smoking, and *ITGA2B* gene expression on platelet function and MACE. Aspirin (1) and smoking (2) have primary, anti- and pro- thrombotic effects on platelet function, respectively. Secondary, compensatory effects of aspirin (3) and smoking (4) on ITGA2B expression are opposite their primary effects on platelet function and act to restore platelet function in a homeostatic manner (5, 6). *ITGA2B* expression represents a component of overall platelet function; higher levels (regardless of their cause) are associated with higher MACE risk. The overall effect of aspirin on MACE is the combination of its primary and compensatory effects (1, 3, and 5), and is protective. The effect of smoking on MACE is the combination of its primary and compensatory effects (2, 4, and 6), and overall increases risk

A primary purpose of this study was to investigate potential interactions between cigarette smoking, platelet function and CV-related events. Unexpectedly, we observed a significant inverse relationship between the sGES and ITGA2B expression levels (Fig. 3); this was also observed between smoking status and ITGA2B expression. One possible explanation for these findings is that the pro-thrombotic effects of smoking on platelets may lead to secondary effects which lower expression of aspirin response genes, including ITGA2B. Under this model (Fig. 4), an activator of platelet function such as smoking leads to a decrease in aspirin response genes in patients with excessive platelet activation, consistent with a homeostatic feedback mechanism that aims to restore platelet function to normal levels. A similar but opposite effect was observed in our prior work where we demonstrated that ITGA2B levels are inversely correlated with platelet aggregation in healthy volunteers on aspirin, with higher levels associated with lower residual platelet aggregation [9]. While speculative, this model proposes that the expression of aspirin response genes in platelets may serve as a “sensor” within a given individual modulating excessively low or high levels of platelet aggregation caused by environmental stimuli. An example of such a homeostatic mechanism exists for maintaining platelet count through the platelet thrombopoeitin (TPO) receptor, which binds circulating TPO. In this system thrombocytopenia leads to higher circulating levels of TPO, which can then stimulate thrombopoesis by the bone marrow. Similarly, thrombocytosis leads to lower TPO levels to reduce platelet production. Other possible explanations for the observed inverse correlation are: 1) that although there is discordance at the mRNA level that there is positive correlation at the protein level; 2) smoking may alter the composition of circulating platelets (by shifting their clearance, production, or maturity, for example) such that high ITGA2B expressing platelets are underrepresented thus creating a synthetic inverse correlation with smoking; or 3) confounding due to some other clinical factor and smoking that lowers ITGA2B expression. In this study increased levels of ITGA2B and the sGES were associated with MACE; these associations may be independent of the homeostatic mechanism described in the above model. The associations of the smoking GES and ITGA2B levels with MACE appear to be independent, as both associations remained significant in a multi-variable model (Table 4) suggesting that the effects of smoking on MACE are not entirely through their effects on platelets. We did not observe an interaction between the GES and ITGA2B (Table 4), however the analysis was likely underpowered to detect such an interaction should it exist.

This study has a number of limitations. Despite the use of subjects referred for invasive angiography, the MACE rate in the population used for this study was only 3.5%, thus limiting our power to detect effects and interactions. Our clinical data regarding smoking status was limited; details regarding the amount or type of cigarette smoke exposure was lacking, and we did not have information about which subjects may have been exposed to second hand smoke. The study utilized previously identified gene expression signatures to evaluate interactions between smoking, platelet responsiveness and MACE; prospectively identifying gene expression signatures involved in these interplays may reveal additional subtleties regarding these interactions. Finally, as we are not able perform platelet function analyses on banked samples, we cannot know the levels of platelet function, the relationships to smoking, aspirin, and MACE in this study.

## Conclusion

We have demonstrated that pro- and anti-thrombotic gene expression signatures are associated with MACE, and that these signatures are independent of each other and with traditional risk factors. Additional work will be necessary to further elucidate the biology behind these interactions and to shed additional light on the relationships between smoking, platelet responsiveness, and increased cardiovascular disease risk.

## Additional files

Additional file 1: Table S2. This file contains the RT-qPCR probe and primer sequences for the 5 genes comprising the sGES. (XLSX 38 kb)

Additional file 2: Table S1. This file contains the primary gene expression data including the sGES (SMOKSCO), ITGA2B gene expression levels (ITGA2BPR), current aspirin use (Aspirin), current smoking status (CurrrentSmoker), whether the subject experienced MACE (Event), age in 5 year bins (AgeBin), platelet count (PLATELET), whether the subject had obstructive CAD (oCAD), and the expression levels for the individual genes comprising the smoking GES. 1 = positive, 0 = negative in all binary fields. (XLSX 192 kb)

Additional file 3: Table S3. This file contains Site, PI, and IRB number information from the PREDICT trial. (XLSX 10 kb)

## Abbreviations

AIC: Aikman Information Criteria
ARS: Aspirin response signature
AUC: Area under the Curve
CVD: Cardiovascular disease
FRS: Framingham Risk Score
MI: Myocardial infarction
oCAD: Obstructive coronary artery disease
OR: Odd Ratio
QCA: Quantitative coronary angiography
ROC: Receiver Operator Characteristic
RT-qPCR: Real time, quantitative Polymerase Chain Reaction
sGES: Smoking gene expression score
TIA: Transient ischemic attack
